# 1330. Characteristics and Outcomes of External Ventricular Drain Infections

**DOI:** 10.1093/ofid/ofad500.1168

**Published:** 2023-11-27

**Authors:** Lucy B Tran, Curtis Converse, Jessica Costales-Cantrell

**Affiliations:** Arrowhead Regional Medical Center, Colton, California; Arrowhead Regional Medical Center, Colton, California; Arrowhead Regional Medical Center, Colton, California

## Abstract

**Background:**

External ventricular drain (EVD)-related ventriculitis is a serious complication associated with poor patient outcomes. We aim to: (1) characterize patient-level factors and outcomes of individuals with EVD-related ventriculitis and (2) identify differences by type of infection and indication of EVD-placement.

**Methods:**

This study is a single center, case series of adults hospitalized with a diagnosis of EVD-related ventriculitis between January 2018 to February 2023. EVD-related ventriculitis was defined as having a positive cerebrospinal fluid culture from an EVD consistent with CDC/NHSN criteria for ventriculitis. Several covariates were extracted from patient charts to characterize patient outcomes. Further stratification by type of infection (gram-positive versus gram-negative ventriculitis) and indication (traumatic versus non-traumatic EVD placement) were performed to identify any differences in characteristics between the two groups.

**Results:**

Twenty-two patients were identified in the study. The majority of patients were infected with gram-positive bacteria (54.5%) rather than gram-negative bacteria (41%) with 1 case of fungus. The most common indication for EVD placement was trauma (59%) and most were placed at bedside (91%) rather than an operating room (9%). The mean length of stay (LOS) was 42.14 days. (Table 1)

There was a statistically significant difference in the CSF to serum glucose ratio between gram-negative and gram-positive infections with gram negative ventriculitis having a lower ratio compared to patients with gram positive ventriculitis (0.23 vs 0.59, p=0.0005). However, LOS, interval from catheterization to infection, and other CSF parameters were similar between the two groups as well as those whose EVDs were placed in the setting of traumatic vs non-traumatic settings. (Table 2 and 3)

**Table 1.**
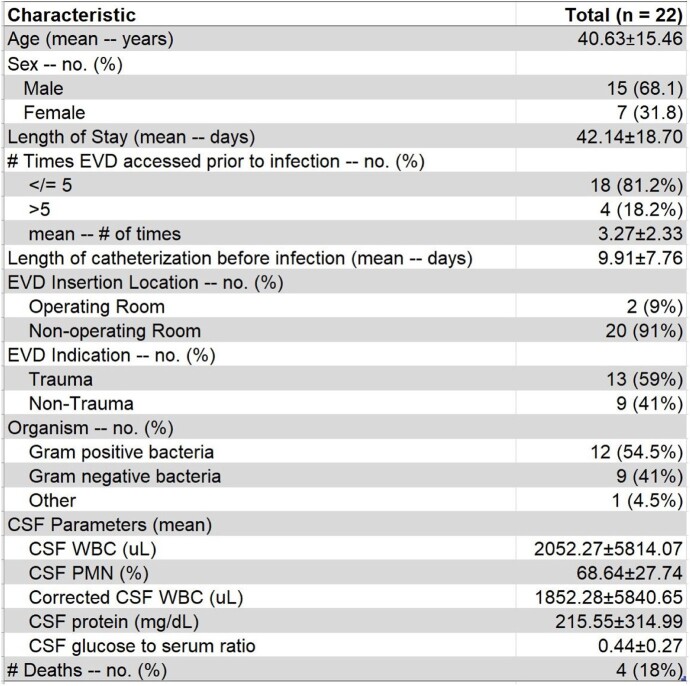
Demographics and characteristics of 22 patients with EVD-associated infections

**Table 2.**
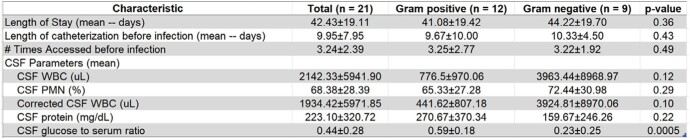
Characteristics stratified by bacterial organism type

Characteristics stratified by trauma vs non-trauma patients

**Figure:**
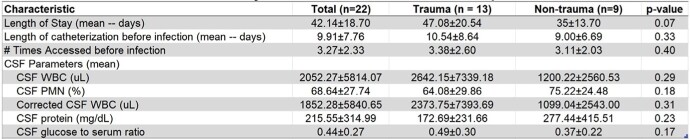

**Conclusion:**

Patients with EVD-related ventriculitis had double the LOS compared to neurosurgical patients without EVD-related ventriculitis reported in the literature. Patients with gram-negative ventriculitis had a lower CSF to serum glucose ratio than those with gram-positive ventriculitis. Otherwise, patient characteristics and outcomes were similar when stratified by type of infection and indication.

**Disclosures:**

**All Authors**: No reported disclosures

